# The association between self-reported nocturnal sleep duration, irregularity in daily energy intake and diet quality in a sample of Iranian adults

**DOI:** 10.1017/S1368980023000502

**Published:** 2023-08

**Authors:** Vida Yazdani, Sheida Zeraattalab-Motlagh, Ahmad Jayedi, Maryam Majdi, Amin Mirrafiei, Fahimeh Martami, Kurosh Djafarian, Sakineh Shab-Bidar

**Affiliations:** 1 Department of Community Nutrition, School of Nutritional Sciences and Dietetics, Tehran University of Medical Sciences (TUMS), Tehran 1416753955, Islamic Republic of Iran; 2 Department of Clinical Nutrition, School of Nutritional Sciences and Dietetics, Tehran University of Medical Sciences (TUMS), Tehran, Islamic Republic of Iran

**Keywords:** Sleep length, Energy intake, Diet quality, Healthy Eating Index-2015

## Abstract

**Objective:**

Evidence on the relationship between sleep duration and irregularity in daily energy intake with diet quality in Iranian adults is scarce. We aimed to evaluate the association of sleep duration with diet quality and irregularity in daily energy intake.

**Design:**

This is a cross-sectional study.

**Setting:**

The study was performed in healthcare centres in Tehran.

**Participants:**

739 adults aged 20–59 years were recruited. Dietary intake was assessed by a FFQ and three 24-h dietary recalls. Diet quality was assessed using the Healthy Eating Index-2015 (HEI-2015). An irregularity score of daily energy intake was calculated based on the deviation from the 3-d mean energy intake. Sleep duration was estimated using self-reported nocturnal sleep duration by each person.

**Results:**

The mean age of the study participants was 44·4 ± 10·7 years; 70 % were women. The mean nocturnal sleep duration, HEI score and irregularity score were 6·7 ± 1·22 h/d, 52·5 ± 8·55 and 22·9 + 19, respectively. After adjusting for potential confounders, sleep duration was not associated with adherence to HEI-2015 (OR: 1·16; 95 % CI 0·77, 1·74). Longer sleep duration was marginally associated with a lower odd of irregularity in daily energy intake. However, after adjustment for various confounders, this association was not significant (OR: 0·82; 95 % CI 0·50, 1·33; *P*_trend_ = 0·45). No significant interaction was observed between sleep duration and irregularity in daily energy intake in relation to adherence to HEI-2015 (*P*_interaction_ = 0·48).

**Conclusions:**

We found that sleep duration was not associated with adherence to HEI-2015 and irregularity in daily energy intake. Further prospective studies are warranted.

Increasing attention has been paid to the potential metabolic implications of inadequate sleep. Several cross-sectional and epidemiological studies have reported that insufficient sleep duration is related to obesity, hypertension, type 2 diabetes, CVD and all-cause mortality^(1–4)^. The exact mechanisms by which sleep restriction may lead to such diseases have not been well established. Therefore, it needs to be clarified whether chronic disease resulted from the curtailment of sleep duration by itself or was a result of other relevant risk factors. Previous studies have suggested that inadequate sleep duration may change dietary intakes^(5)^, reduce physical activity^(6)^ and modify orexigenic and anorexigenic hormone levels^(7)^.

Time of eating and irregularity in energy intake are other possible related mechanisms, linked to lower diet quality. A greater daily variation in energy intake, described as a higher irregularity score of daily energy intake, is associated with a lower Healthy Eating Index (HEI-2015) score^(8)^. Regulation of energy intake occurs through short-term signals that control hunger, food intake and satiety, and through long-term signals that relate to the conservation of energy stores, lean tissue or both. Traditional nutritional research has primarily focused on single nutrients or food groups^(5,9,10)^, and a limited number of studies examined the link between sleep duration and diet quality^(11)^, which can provide a comprehensive insight into this context due to the complexity of the overall diet. Sleep duration may be a risk factor for poor diet quality. In a recent study, higher intake of energy, fat and carbohydrate has been reported among short sleepers than among longer sleepers^(6)^. Findings from a cross-sectional study reported that females with a long sleep duration had a higher quality of diet, higher intake of some nutrients and better weight status^(11)^. In another newly conducted study, a U-shaped association between sleep duration and eating behaviours was observed among middle-aged and older women; women with short or long sleep duration were more likely to eat during unconventional hours and replace meals with snacks than women with adequate sleep duration^(12)^. Sleep deprivation occurs frequently among women with young children, and short sleep duration has been associated with poor diet quality among young women, including skipped meals, higher energy intake from snacks and beverages and greater sugar consumption^(13)^.

Recent studies have shown that more day-to-day variation in energy intake was associated with a higher likelihood of adiposity^(14)^ and a higher risk of metabolic syndrome^(15)^ which may be correlated with lower diet quality. Moreover, it is reported that people with a short sleep duration may have low adherence to a healthy diet and regular meal patterns^(16)^. A study by Dashti *et al*. also showed that short sleep duration was associated with increased odds of irregular eating behaviour and unbalanced food variety^(17)^. As previously indicated, short sleep duration and meal irregularity especially energy irregularity have been identified as important factors in the low-quality diet which may lead to the burden of disease. As a result, it is hypothesised that longer sleep duration and less daily variation in energy intake have the potential to be associated with adherence to a healthy diet. Although many studies evaluate sleep duration and energy intake, obesity and consumption patterns for some food groups, the association of nocturnal sleep duration with the HEI-2015 and irregularity in daily energy intake has not been investigated in Iran. Therefore, this cross-sectional study aimed to evaluate the potential association of sleep duration with diet quality and irregularity in daily energy intake in a group of Iranian adults.

## Methods

### Study participants

In the present cross-sectional study, 850 men and women, aged between 20 and 59 years, from different healthcare centres in five areas of Tehran, Iran, were recruited from 2018 to 2019. This is a secondary analysis of a dataset of our previous study that initially was planned to find the association between meal-based dietary patterns and obesity^(18)^. Then, considering the total prevalence of overweight and obesity of 65 % in Tehran adults^(19)^, with a maximum estimation error of 5 % and a value of d of 0·04, the sample size was obtained using the following formula: n = z2.p (1-p)/d2 = 546. Since the sampling method is clustered and classified into two stages, and the response rate of individuals in each health centre may be correlated, the number of samples was multiplied by the effective coefficient of the cluster design of 1·5, leading to a total number of 819 participants. Finally, we could recruit 850 participants for the present study. A two-stage cluster sampling method was used for the recruitment of participants with an equal number of subjects selected from each health centre by a simple random sampling method. Eligible participants for inclusion in the study were seemingly healthy adults 20–59 years, residing in Tehran, who were present at local healthcare centres during the study period and were willing to participate in the study. Pregnant and lactating women were not included in the study. After excluding participants who had at least one uncompleted variable, 739 subjects were involved in the study.

### Socio-demographic data

Information on age, sex, education (illiterate, under diploma = uncompleted primary or secondary education, diploma = completed secondary education, educated = bachelor’s degree or higher), marriage (single or married), smoking status (never smoker or former smoking or current smoking) and occupation (employed or housekeeper or retired or unemployed) was obtained from each participant during a private interview by a trained interviewer. Information on diabetes and history of hypertension was extracted from the health records of the patients.

### Physical activity

Physical activity was assessed using seven items in the short form of the International Physical Activity Questionnaire –Short Form questionnaire, which asked about the frequency and duration of vigorous intensity, moderate intensity and walking physical activity^(20)^. These data were summarised to report physical activity in categories, classifying populations into high-, moderate- and low-active groups. Time spent in vigorous, moderate and walking activity was weighted by the energy expended for these categories of activity, to produce MET-minutes of physical activity. MET were classified as low (< 600 MET-minutes/week), moderate (600–3000 MET-minutes/week) and vigorous (> 3000 MET-minutes/week).

### Anthropometric assessment

Participants’ body weight was measured by using a digital scale with a sensitivity of 0·1 kg (808Seca), with a light cloth and without wearing shoes. Height was measured by using a wall stadiometer with 0·1 cm precision (Seca) while the subjects were standing without shoes. Measurement of waist circumference was performed by using a flexible anthropometric tape between the iliac crest and lower rib margin with 0·1 cm accuracy. Hip circumference was measured using a flexible anthropometric tape with an accuracy of 0·1 cm, in the correct horizontal location at the maximum level from the lateral facet over thin clothing without any pressure on the body surface. BMI was calculated by dividing the body weight in kg by height in metres squared.

### Dietary assessment

Dietary intake was evaluated by using a validated 168-item semi-quantitative FFQ and three 24-h dietary recalls^(21)^. Trained dietitians completed the FFQ and the first 24-h dietary recall by face-to-face interview during the first visit to each health centre. The other two 24-h dietary recalls were completed on random days including one weekend, by telephone interviews. The portion sizes of the foods were converted to grams per day using household measures. Intake of energy and nutrients was estimated using Nutritionist IV software, based on the US Department of Agriculture food composition database modified for Iranian foods^(22)^.

### Calculating score of irregularity in daily energy intake

To evaluate day-to-day variations in total daily energy intake for each participant, a new-developed irregularity score, introduced by Pot *et al*.^(14)^, was calculated. First, the absolute difference of the individual daily energy intake from the 3-d mean energy intake was divided by the 3-d mean energy intake. Then, these values were multiplied by 100 and then averaged over the 3 d. This score served as a proxy for irregularity in daily energy intake. Accordingly, a low score indicates a smaller day-to-day variation in daily energy intake and more regular daily energy intake and a high score indicates a larger day-to-day variation in daily energy intake.

### Diet quality

The overall quality of the diet was evaluated by using the HEI-2015. The HEI-2015 score measures the degree of adherence to the 2015–2020 US Dietary Guidelines for Americans and ranges from 0 to 100 points^(23)^. Healthy components that received points for higher consumption included total fruits, whole fruits, total vegetables, greens and beans, whole grains, dairy products, total protein, seafood and plant proteins and the ratio of unsaturated fat to saturated fat. Unhealthy components that received points for lower consumption included refined grains, saturated fat, Na and added sugar. We did not include whole grain and Na intake in the score due to the lack of consumption of whole grains in the Iranian diet and the fact that dietary salt intake was not recorded in the FFQ. Therefore, the possible minimum and maximum scores are, respectively, 0 and 80 (compared with 0 and 100 in the traditional HEI-2015). Adequacy components are different dietary elements that are encouraged for consumption, and higher scores reflect higher intake. Moderation components represent dietary elements that are recommended to be limited, and higher scores reflect lower intake. Adequacy components include total fruit (0–5 points), whole fruits (0–5 points), total vegetables (0–5 points), greens and beans (0–5 points), whole grains (0–10 points), dairy products (0–10 points), total protein foods (0–5 points), seafood, and plant proteins (0–5 points) and fatty acids (0–10 points). Moderation components include refined grains (0–10 points), Na (0–10 points), added sugars (0–10 points) and saturated fats (0–10 points). Each component is given a score and then summed to generate a total HEI-2015 score. The method of scoring and definition of HEI-2015 in our study are presented in online supplementary material, Supplemental Table 1.

### Sleep duration assessment

Information about the nocturnal sleep duration was obtained from participants using self-report, as we asked all participants the question: ‘How many hours do you normally sleep during the night?’

### Statistical analysis

The sleep duration was categorised into tertiles, and characteristics of the study participants were reported across the tertiles of the sleep duration. Demographic characteristics of the participants across the tertiles of the score were compared using *χ* ^2^ for categorical variables and the ANOVA test for continuous variables. Intake of macronutrients and food groups was expressed as mean and sd and compared using the ANOVA test. Binary logistic regression analysis was used to investigate the association of sleep duration with adherence to HEI-2015 in crude and multivariable-adjusted models, based on the median of the HEI score (=53) as the cut-off. The scores of the irregularity in energy intake were also categorised into tertiles. We used ordinal logistic regression to estimate OR (95 % CI) for having higher irregularity scores in daily energy intake by increasing the sleep duration in crude and multivariable-adjusted models. In both binary logistic regression and ordinal logistic regression, the following models were used to control confounding variables: In model 1, adjustment was made for age, sex and energy intake (except for ordinal logistic regression). Model 2 was additionally adjusted for marital status, education, disease history (diabetes, hypertension and hyperlipidaemia) and occupation. Additional adjustment in model 3 was conducted for physical activity and smoking status. In the final model, we also adjusted for BMI. We also used two-way ANOVA to investigate the combined association of sleep duration and irregularity in daily energy intake on the quality of diet. All analyses were carried out using SPSS software (SPSS Inc., version 22), and *P* < 0·05 was defined as significant.

## Results

This cross-sectional study included 850 adults, including 584 women and 266 men aged 20–59 years, with a mean age of 44·7 ± 10·8 years and a mean BMI of 27·9 ± 5·6 kg/m^2^. The mean nocturnal sleep duration, HEI score, and irregularity score were 6·7 + 1·22 h/d, 52·5 + 8·55 and 22·9 + 19, respectively.

General characteristics of the study population across the tertiles of sleep duration are presented in Table 1. Individuals in the top tertile of sleep duration had higher physical activity and lower waist circumference and were more likely to be married and hypertensive. No other significant difference was found for other characteristics across the tertiles of sleep duration.

**Table 1:** General characteristics of participants across the tertiles of sleep duration

	T_1_ (< 6 h)		T_2_ (6–7 h)		T_3_ (> 7 h)		
	Mean	sd	Mean	sd	Mean	sd	P*
Age (year)	10·9	44·7	10·8	44·0	10·4	44·5	0·71
BMI (kg/m^2^)	4·69	27·9	4·86	27·7	4·52	27·7	0·86
HEI-2015 score	52·3	8·67	53·4	8·28	52·0	8·41	0·17
Adhering to HEI-2015							0·81
25–49							
*n*	91		75		79		
%	37·1		30·6		32·2		
50–56							
*n*	76		76		68		
%	34·5		34·5		30·9		
57–77							
*n*	91		91		75		
%	35·4		35·4		29·2		
Irregularity score							
Mean	24·7		23·1		20·8		0·10
sd	21·6		18·5		18·0		
Sex (male) (%)	33·1		33·3		25·6		0·11
Waist circumference (cm)							
Mean	11·4		13·4		12·5		0·04
sd	93·4		91·1		90·9		
	%		%		%		
Marital status (single) (%)	77·9		85·1		81·1		0·03
Education (educated) (%)	35·7		34·9		31·7		0·05
Hypertension (yes) (%)	14·4		12		21·6		0·01
Diabetes (yes) (%)	8·7		7·3		9·7		0·63
Physical activity (inactive) (%)	84		69·1		44·9		0·001>
Smoking (smoker) (%)	6·1		6·4		3·1		0·50

Data are presented as mean (sd) or percentage.

HEI, Healthy Eating Index.

*Obtained from ANOVA or *χ* ^2^, where appropriate.

Dietary intakes of participants across the tertiles of sleep duration are indicated in Table 2. No significant differences were observed in terms of dietary intakes across the tertiles of sleep duration.

**Table 2:** Dietary intakes of participants across the tertiles of sleep duration

	*T*_1_ (< 6 h)		T_2_ (6–7 h)		T_3_ (> 7 h)		
Sleep duration	Mean	se	Mean	se	Mean	se	P*
Energy (kJ/d)	10 376	274	10 669	364	11 593	928	0·28
Protein (g/d)	2·23	83·7	2·9	87·0	4·2	91·8	0·19
Fat (g/d)	3·01	80	3·56	84·1	3·38	83·3	0·76
Carbohydrate (g/d)	9·93	363	11·4	374	56·0	434	0·23
SFA (g/d)	1·09	25·1	1·01	24·4	1·25	25·1	0·87
MUFA (g/d)	1·18	25·5	1·33	25·6	2·93	27·2	0·77
PUFA (g/d)	0·64	16·6	0·93	17·5	0·92	17·6	0·63
Fruit (g/d)	21	371	39·3	410	18·3	352	0·33
Vegetables (g/d)	21·4	446	22·6	434	23	399	0·31
Dairy products (g/d)	30·3	507	40·4	557	28·6	469	0·19
Grains (g/d)	17·6	425	21·9	441	15·3	409	0·50
Nuts (g/d)	1·64	14·6	1·80	14·9	3·33	19·4	0·27
Meats (g/d)	7·78	111	5·60	112	5·11	102	0·49

Data are presented as mean (s e) or percentage.

*Obtained from one-way ANOVA,

Multivariable-adjusted OR and 95 % CI for HEI-2015 across categories of sleep duration are presented in Table 3. Compared to those with the lowest sleep duration, individuals with the highest sleep duration had no greater odds of adherence to HEI-2015 (OR: 1·12; 95 % CI 0·87, 1·62). Such a non-significant association was also seen after controlling for potential confounders (OR: 1·16; 95 % CI 0·77, 1·74).

**Table 3:** Multivariable-adjusted OR and 95 % CI of adherence to HEI-2015 (median score of 53) across the tertiles of sleep duration

	Tertiles of sleep duration					
		*T*_2_ (6–7 h)		*T*_3_ (> 7 h)		
	*T*_1_ (< 6 h)	OR	95% CI	OR	95% CI	*P*_trend_*
Crude	1	1·34	1·91–0·94	1·12	1·62–0·87	0·47
Model 1	1	1·38	1·98–0·96	1·18	1·71–0·81	0·34
Model 2	1	1·43	2·08–0·98	1·16	1·71–0·79	0·39
Model 3	1	1·43	2·09–0·98	1·16	1·74–0·77	0·41
Model 4	1	1·42	2·09–0·97	1·16	1·74–0·77	0·41

HEI, Healthy Eating Index.

Data are presented as OR and 95 % CI.

Low adherence to HEI-2015 ≤ 53.

Model 1: Adjusted for age, gender and energy intake.

Model 2: Additionally, adjusted for marital status, education, disease history (diabetes, hypertension and hyperlipidaemia) and occupation.

Model 3: Additional adjustment for physical activity and smoking.

Model 4: Additional adjustment for BMI.

*Obtained from binary logistic regression

Table 4 shows the crude and multivariable-adjusted OR (95 % CI) for being in higher tertiles of irregularity in daily energy intake scores across sleep duration categories. In the crude model, long sleep duration was marginally associated with a lower odd of irregularity in daily energy intake (OR: 0·64; 95 % CI 0·41, 1·00). However, after adjustment for various confounders, this association was not significant (OR: 0·82; 95 % CI 0·50, 1·33).

**Table 4:** Multivariable-adjusted OR and 95 % CI of irregularity score^1^ in dalily energy intake across the tertiles of sleep duration

	Tertiles of sleep duration					
		*T*_2_ (6–7 h)		*T*_3_ (>7 h)		
Tertiles of irregularity score in daily energy intake	*T*_1_ (<6 h)	OR	95% CI	OR	95% CI	*P*_trend_*
Crude						
T1 (<12·3)	1	1		1		
T2 (12·3–23·8)	1	0·75	0·49–1·17	0·77	0·50–1·20	0·24
T3 (>23·8)	1	0·86	0·56–1·32	0·64	0·41–1·00	0·05
Model 1						
T1 (<12·3)	1	1		1		
T2 (12·3–23·8)	1	0·74	0·48–1·15	0·79	0·51–1·23	0·28
T3 (>23·8)	1	0·85	0·55–1·30	0·63	0·40–1·00	0·05
Model 2						
T1 (<12·3)	1	1		1		
T2 (12·3–23·8)	1	0·73	0·46–1·13	0·79	0·50–1·24	0·29
T3 (>23·8)	1	0·85	0·55–1·31	0·64	0·40–1·01	0·06
Model 3						
T1 (<12·3)	1	1		1		
T2 (12·3–23·8)	1	0·81	0·51–1·27	1·01	0·62–1·62	0·99
T3 (>23·8)	1	0·94	0·61–1·47	0·82	0·50–1·33	0·45
Model 4						
T1 (<12·3)	1	1		1		
T2 (12·3–23·8)	1	0·81	0·51–1·27	1·01	0·62–1·62	0·99
T3 (>23·8)	1	0·94	0·61–1·47	0·82	0·50–1·33	0·45

Data are presented as OR and 95 % CI.

^1^Irregularity score: higher score indicates higher irregularity in energy intake.

Model 1: Adjusted for age, gender and energy intake.

Model 2: Additionally, adjusted for marital status, education, disease history (diabetes, hypertension and hyperlipidaemia) and occupation.

Model 3: Additional adjustment for physical activity and smoking.

Model 4: Additional adjustment for BMI.

*Obtained from ordinal logistic regression model.

Results for the interaction between sleep duration and irregularity in daily energy intake with adherence to HEI-2015 are shown in Table 5. No significant interaction was observed between sleep duration and irregularity in daily energy intake on adherence to HEI-2015 (*P*_interaction_ = 0·48).

**Table 5:** The interaction between sleep duration and irregularity score^1^ in daily energy intake with mean score of adherence to HEI-2015

	Tertiles of sleep duration						
	T_1_ (<6 h)		T_2_ (6–7 h)		T_3_ (>7 h)		
Tertiles of irregularity score in daily energy intake	Mean	se	Mean	se	Mean	se	*P*_interaction_*
T1 (<12·3)	53·2	0·99	53·2	0·96	52·7	0·95	0·48
T2 (12·3–23·8)	51·7	0·87	53·8	0·97	52·4	0·95	
T3 (>23·8)	52·0	0·88	53·0	0·89	50·6	1·05	

HEI, Healthy Eating Index.

HEI-2015 is presented as mean ± se.

^1^Irregularity score: higher score indicates higher irregularity in energy intake.

*P* value derived from the two-way ANOVA.

## Discussion

In this cross-sectional study, even after controlling for potential confounders, no significant association was observed between sleep duration and adherence to HEI-2015. Longer sleep duration was marginally associated with a lower odds of irregularity in daily energy intake that disappeared after adjustment for various confounders. Moreover, according to the interaction analysis that we conducted, sleep duration and irregularity in daily energy intake were not associated with adherence to HEI-2015.

Sleep duration and diet are two important parts of the lifestyle. There is evidence that the length of sleep is a risk factor for overweight and obesity^(24)^. Short sleep duration can affect obesity by increasing or altering food intake^(25)^. Therefore, sleep as a modifiable factor can play a major role in improving the diet and preventing various diseases. Most observational studies investigating the relationship between sleep duration and dietary intake have examined individual nutrients, food groups or energy intake, while people do not consume a single nutrient or food group. Due to the complexity of the diet and the interaction of the food components with each other, addressing the multidimensional nature of the diet provides better insight. Diet indices are the best approach for this purpose. On the other hand, daily variations in energy intake are also associated with poor diet quality and metabolic diseases^(16,17)^. To our knowledge, this is the first study that examined the association of sleep duration with HEI-2015 and irregularity in energy intake in Iranian adults.

In the current study, we found that sleep duration was not associated with adherence to HEI-2015. In contrast to our findings, short sleep duration combined with poor sleep quality is associated with low adherence to a healthy diet and regular meal patterns in a large prospective cohort study^(16)^. Bel *et al*. reported that short sleep duration was associated with lower dietary quality in European adolescents^(26)^. A similar finding was also seen in a cross-sectional study in which short sleepers had significantly lower diet quality indices and a higher percentage of body fat and abdominal adiposity compared with longer sleepers, without any significant differences in physical activity levels^(11)^. These inconsistent results might be explained by differences in study populations, methods used to assess dietary intakes, indices that are applied to determine the quality of the diet and also differences in the cut-off points that are used to define sleep categories. Moreover, differences in analytic approaches could be another reason for the conflicting results of various studies. For example, factors such as anxiety and depression can influence the quality of diet and sleep duration^(27,28)^. Lack of adjustment for such confounders can affect the independent association between sleep duration and diet quality.

In the current study, higher sleep duration was marginally associated with lower odds of irregularity in daily energy intake. However, after adjustment for various confounders, this association was not significant. In a study, short-term sleep loss neither increased food intake nor affected concentrations of the hunger-regulating hormones leptin and ghrelin^(6)^. Findings from a cross-sectional study indicated that short-duration sleepers began eating earlier and ended their eating later in the day, but despite the longer eating period, they did not report more eating events. Moreover, the total daily energy intake was not related to sleep duration^(29)^. Kim *et al*. reported that women with long (> 10 h) sleep duration had eating patterns similar to those with short (< 6 h) sleep duration. A lower tendency for eating during conventional eating hours and greater snack dominance over meals was also related to higher intakes of fat and sweets for energy and lower intakes of fruits and vegetables^(12)^. One reason underlying these differences might derive from the circadian clock disruption. Indeed, the circadian system plays a prominent and direct role in regulating sleep, energy metabolism and feeding^(30,31)^. For example, leptin, a hormone that is regulated by circadian rhythms, decreases appetite, increases energy expenditure and influences sleep efficiency^(32)^. It has been shown recently that a disrupted circadian clock leads to reduced serum levels of leptin independent of sleep duration. Another case that can be mentioned is the measurement errors in estimating dietary intakes, which can explain the differences in the findings of various studies^(33)^.

In the present study, the association of sleep duration and irregularity in daily energy intake with adherence to HEI-2015 was not significant. No study examined the interaction between sleep duration and irregularity in daily energy intake with diet quality, so the findings of the present study are not comparable to similar previous studies. St-Onge *et al*. reported that there were no effects of sleep timing, meal timing or their interaction on carbohydrate, protein, fibre, sugar or Na intakes. The sleep timing × meal timing interaction was significant for energy and monounsaturated fat intakes only^(34)^. Further studies are needed to examine this association.

This study has several strengths. First, a relatively large sample size was included in our analysis. Second, the availability of comprehensive information on dietary and non-dietary covariates allowed us to control for a wide range of potential confounders to obtain an independent association. Furthermore, validated questionnaires were applied for data collection, which can further support the accuracy of the findings. However, some limitations need to be considered. First, the causality of the associations cannot be established from this study. Second, although we controlled for most lifestyle factors and diet quality, residual or unmeasured confounding cannot be excluded due to the observational nature of the study. Third, as is usually the case in nutritional epidemiology, misclassification of study participants due to the use of FFQ is unavoidable.

## Conclusion

We found that sleep duration was not associated with adherence to HEI-2015 and irregularity in daily energy intake. Further studies, specifically with prospective design, are warranted.

## Supplementary material

For supplementary material accompanying this paper visit https://doi.org/10.1017/S1368980023000502. click here to view supplementary material
