# Supplementary material for: The association between self-reported nocturnal sleep duration, irregularity in daily energy intake and diet quality in a sample of Iranian adults
Source: Public Health Nutr. 2023 Apr 11;26(8):1609–16. doi: 10.1017/S1368980023000502 (PMC10410366; doi:10.1017/S1368980023000502)
Supplement: Supplementary file 1 [file S1368980023000502sup001.docx]

**Supplementary Table 1.** The method of scoring and definition of HEI-2015

| Component | Maximum Scores | Standard for Maximum Scores | Standard for Minimum of zero |
| --- | --- | --- | --- |
| Adequacy |  |  |  |
| Total fruits | 5 | $\geq$0.8 cup | No fruits |
| Whole fruits | 5 | $\geq$0.4 cup | No Whole fruits |
| Total vegetables | 5 | $\geq$ 1.1 cup | No vegetables |
| Greens & Beans | 5 | $\geq$ 0.2 cup | No Greens & Beans |
| Whole Grains | 10 | $\geq$ 1.5 Oz | No Whole Grains |
| Dairy | 10 | $\geq$ 1.3 cup | No Dairy |
| Total Protein Foods | 5 | $\geq$ 2.5 Oz | No Protein Foods |
| Seafood & plant proteins | 5 | $\geq$ 0.8 cup | No Seafood & plant proteins |
| Fatty Acids Moderation | 10 | $(PUFAs+MUFAs)/SFAs\geq$ 2.5 | $(PUFAs+MUFAs)/SFAs$ $\leq$1.2 |
| Refined Grains | 10 | $\leq$1.8 Oz | $\geq$ 4.3 Oz |
| Sodium | 10 | $\leq$ 1.1 g | $\geq$ 2.0 g |
| Added Sugars | 10 | $\leq$ 6.5% of energy | $\geq$ 26% of energy |
| Saturated Fats | 10 | $\leq$ 8% of energy | $\geq$ 16% of energy |
